# Trends and Thematic Clusters in Studies on Biosensors for Groundwater Quality

**DOI:** 10.3390/bios16100568

**Published:** 2026-10-10

**Authors:** Leonardo Pinto de Magalhães, Ana Carolina de Sousa Silva, Tamara Maria Gomes, Mary Ingrid Alves e Santos, Ernane José Xavier Costa

**Affiliations:** 1 Faculty of Animal Science and Food Engineering (FZEA), University of São Paulo, Pirassununga 13635-900, Brazil; leonardo.magalhaes@alumni.usp.br (L.P.d.M.); tamaragomes@usp.br (T.M.G.); 2 Luiz de Queiroz College of Agriculture (ESALQ), University of São Paulo, Piracicaba 13418-900, Brazil; maryingrid@usp.br

**Keywords:** electrochemical, emerging contaminants, pesticides, arsenic, pollutant detection, food production

## Abstract

Groundwater plays an important role in food production. However, it faces environmental pressures from various factors, and the presence of contaminants in these waters—whether emerging or traditional—is a growing cause for concern. Nevertheless, there are few reviews available that address the analysis of biosensors for the detection of these specific contaminants in groundwater. Therefore, the objective of this study was to conduct a review to determine which types of biosensors, which contaminants, and other factors are most frequently highlighted in studies on the detection of contaminants in groundwater. In line with this objective, the findings indicate a predominance of electrochemical biosensors, with a particular emphasis on the use of bacteria as the biological component. The detection of pesticides has been a focus since the earliest publications, yet there are still significant gaps regarding the detection of multiple substances, such as the co-occurrence of arsenic and other pollutants. Thus, this review contributes to a broad overview of the technologies used, offering important insights for future research and mapping the current state of the art in the detection of contaminants in groundwater.

## 1. Introduction

The use of biosensors for groundwater monitoring is a topic that has not yet been fully explored, presenting significant gaps that must be addressed to improve the quality of this water resource. Existing reviews either fail to systematize the literature (such as by identifying clusters), focus on contaminants in diverse aquatic environments, or analyze biosensor use for environmental monitoring in a general sense. Analyzing their application in groundwater monitoring can enhance the efficiency of treatment and consumption processes, identify avenues for future research, and provide specific context regarding the current state of the art in this field. Given the importance of groundwater in food production, this knowledge also contributes to safer production practices and aids in the conservation of this finite natural resource.

Population growth, as well as technological and economic development in recent decades, has been accompanied by a significant increase in the amount of pollutants discharged into water bodies. This fact represents one of the greatest challenges to human health and to water treatment systems [1]. For example, so-called emerging contaminants have gained prominence in recent years, as they may pose a risk to the ecosystem and are not included in routine environmental monitoring analyses [2]. These contaminants can be of anthropogenic or natural origin, or they can be microorganisms [3]. Furthermore, they occur in the ecosystem at very low concentrations, in the range of parts per trillion (ppt or ng/L) or parts per billion (ppb or μg/L) [4]. Therefore, contaminants are defined as “emerging” if they originate from a new source, require new treatment approaches, or follow a new pathway to reach humans [5].

These contaminants have been found at every stage of the water cycle, from groundwater to wastewater treatment plants [3]. They can pose risks to human health as well as to animals and plants. Consequently, there has been growing concern about how to detect and treat (or reduce) water contaminated by these substances. Among the emerging contaminants, the following stand out: hormones, pesticides, microplastics, pharmaceuticals and personal care products, illicit drugs, and disinfection byproducts, among others [6].

Despite the development of various techniques for detecting these pollutants, some of them remain highly costly [7]. Sensors used in conjunction with new technologies, such as smartphones, can facilitate the use and operation of this equipment for collecting data in the field [8]. Chen et al. [9] note that “future sensor development should focus on eco-friendly materials and user-friendly interfaces.” These authors emphasize that the use of biodegradable materials and smartphone apps can increase the use and acceptance of monitoring sensors for detecting emerging pollutants in water.

Groundwater contamination with these pollutants warrants constant attention, as household products—such as disinfectants, cosmetics, and personal care products—have been detected in high concentrations in groundwater, suggesting that these pollutants accumulate and migrate readily in these environments [10]. Agricultural chemicals, such as organophosphates, are also found in high concentrations in groundwater in developing countries such as Brazil [11]. In these countries, the lack of waste management and low levels of waste treatment, combined with industrial expansion and increased consumption of household chemicals, lead to a rise in the presence of emerging contaminants in water [12].

Protecting groundwater from contamination is a global concern [13]. The increasing demand for food has put pressure on water resources, with approximately 38% of the world’s irrigation water coming from groundwater [14]. In addition to its economic importance for food production, groundwater also has social significance, directly impacting the marketing of food by smallholder farmers in different regions of the world, such as in eastern India [15].

In addition to environmental impacts and risks to human, animal, and plant health, the decline in biocapacity (the ability of ecosystems to regenerate) affects countries’ ability to overcome poverty [16]. Continuous monitoring, with the collection of consistent data, facilitates the assessment of groundwater quality and supports public policy. However, most developing countries lack data—both in terms of quantity and quality—on groundwater contamination, with a lack of both temporal and spatial scale [17] in the collection of such data. This directly impacts these countries’ development and their ability to overcome historical inequalities.

To increase these countries’ access to environmental sensing technologies, the use of biosensors emerges as a promising alternative. Trinh et al. [18], for example, point out that the use of biosensors combined with microfluidic devices offers advantages in environmental monitoring, such as “low cost, selectivity, sensitivity, high automation, and savings in samples and reagents.” In this regard, another important aspect is the integration of biosensors with open-source hardware, a combination that has democratized access to advanced detection technology [19], enabling greater in situ data collection. In the context of groundwater monitoring, this aspect offers advantages over traditional analytical systems, as biosensors enable continuous on-site monitoring, providing an early-warning system and immediate information on the fate and transport of contaminants [20,21]. In addition, the use of these sensors reduces sample preparation time and the use of reagents for this purpose [22], which helps minimize the environmental impacts of the analyses.

In addition to being used to detect emerging pollutants, biosensors are also employed to detect traditional pollutants that have a significant impact on groundwater contamination, such as certain metals, including arsenic [23]. Uranium and arsenic are contaminants associated with various human activities, such as mining [24]; despite the emergence of new sources of contamination, these contaminants still warrant attention in monitoring and collecting data on their levels in groundwater.

To determine which topics the articles in this field address, three guiding questions were defined to help establish and expand upon this review.

Q1—What biological elements are used to monitor emerging pollutants in groundwater? Do they differ from the monitoring of traditional contaminants?
Q2—Which pollutants receive the most attention, and what gaps still need to be filled in the analyses?
Q3—What types of biosensors are most commonly used, and what are their limitations?

## 2. Materials and Methods

To identify the studies included in the review, searches were conducted in the Web of Science (Clarivate Analytics, London, United Kingdom) and Scopus (Elsevier, Amsterdam, Netherlands) databases. The search queries included the following terms, which were entered in the “Topics” field (Web of Science) and in titles, abstracts, and keywords (Scopus):

1. (groundwater* OR “groundwater*” OR “groundwater*” OR “subsurface water*” OR “phreatic water*” OR aquifer) AND;
2. (“emerging pollutant*” OR “Emerging Contaminant*” OR “Contaminants of Emerging Concern” OR “micropollutant*” OR “micropollutant*” OR “unregulated contaminant*” OR “new pollutant*” OR “microplastic*” OR “pesticide*” OR “industrial chemical*” OR “medication*” OR antibiotic* OR “perfluorinated compound*”) AND;
3. (“biosensor*” OR “biological sensor*” OR “biodetector*” OR “biodetector*” OR “bioreporter*” OR “biosonde*” OR OR “bioanalytical device*”).

No restrictions were placed on the publication dates of the articles; the search was limited only to articles published in English (or those with abstracts in that language). The same criteria were applied to both databases to ensure that the results were comparable. In subsequent stages, the analysis was limited to scientific articles (excluding conference papers, reviews, and other publications not classified by the platforms as journal articles).

The search was conducted on 10 June 2026, and the results included articles published up to 2026. Initially, 67 documents were found in Web of Science (of which 45 were articles) and 75 documents (46 articles) in Scopus. After excluding publications that were not journal articles, the combined dataset contained 91 documents. Subsequently, duplicate articles were removed from the combined data (16 articles were excluded), leaving 75 articles for analysis.

The Scopus and Web of Science databases were chosen because, as noted by Olson and Bae [25], the PubMed database has the following disadvantages: lack of citation information; publications on biosensors applied in the biomedical field are given priority; the number of entries included in this database (on biosensors) is limited compared to other databases, such as Web of Science or Scopus; and, in some cases, the metadata is less complete for certain entries.

**Figure 1:**
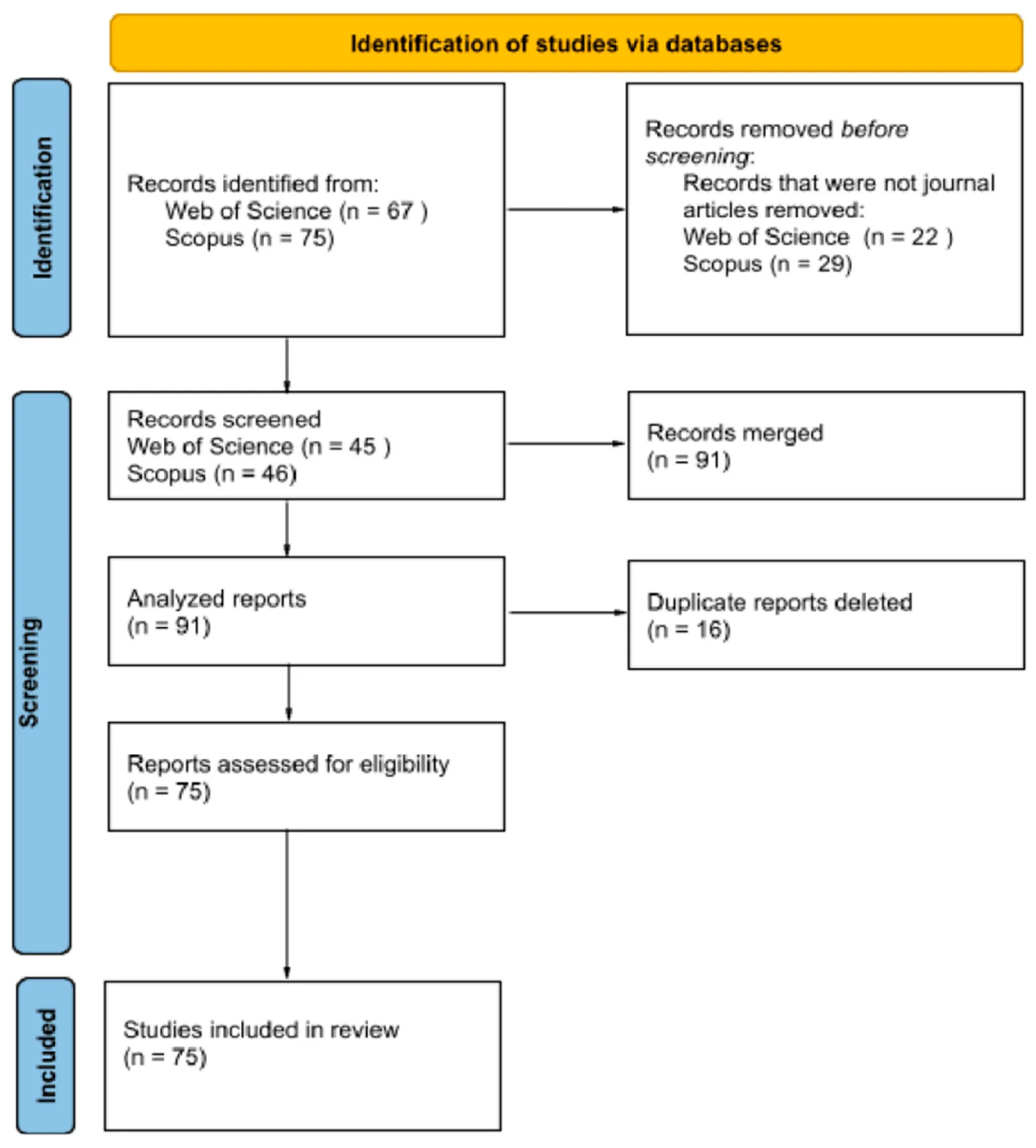
PRISMA flowchart of bibliometric review.

Using the bibliometrix library in RStudio (RStudio, version 4.2.2, Boston, MA, USA), data were obtained on publications regarding the year, the most-cited articles, the most-cited authors, output by country, the journals with the highest output, and the most-cited keywords, as well as trending topics. In addition, a word cloud was generated using the authors’ keywords, along with data on publications by continent, the number of articles by biosensor type, by biological substance used, and by contaminant evaluated. A summary of the steps taken is shown in Figure 1.

To guide the analyses and complement the information provided by bibliometric tools, the articles were analyzed in full (abstract, methodology, and results) to validate the data and add new information to the discussion.

To analyze the set of themes within the group of articles, a clustering process was performed based on the keywords defined by the authors. Using the bibliometrix library, keywords were extracted from each article and compiled into a single table. Subsequently, a presence matrix was created to map which words belonged to which clusters. As a next step, the similarity distance between words was calculated using the ‘binary’ method; in this method, the presence or absence of specific terms was coded as 1 or 0, respectively.

To measure semantic similarity and contextual proximity between terms, a binary distance function based on the Jaccard dissimilarity coefficient was applied. This method quantifies the proportion of asymmetry between variables—disregarding joint absences (0-0 pairs)—so that the distance is computed strictly based on the simultaneous or exclusive presence of words within the same text segments.

The resulting distance matrix served as input for a hierarchical clustering algorithm. This procedure enabled the identification and delineation of word clusters with high association density, revealing the latent thematic structure and the key conceptual cores emerging from the analyzed corpus. The cluster library was used to perform the clustering.

## 3. Results

**Figure 2:**
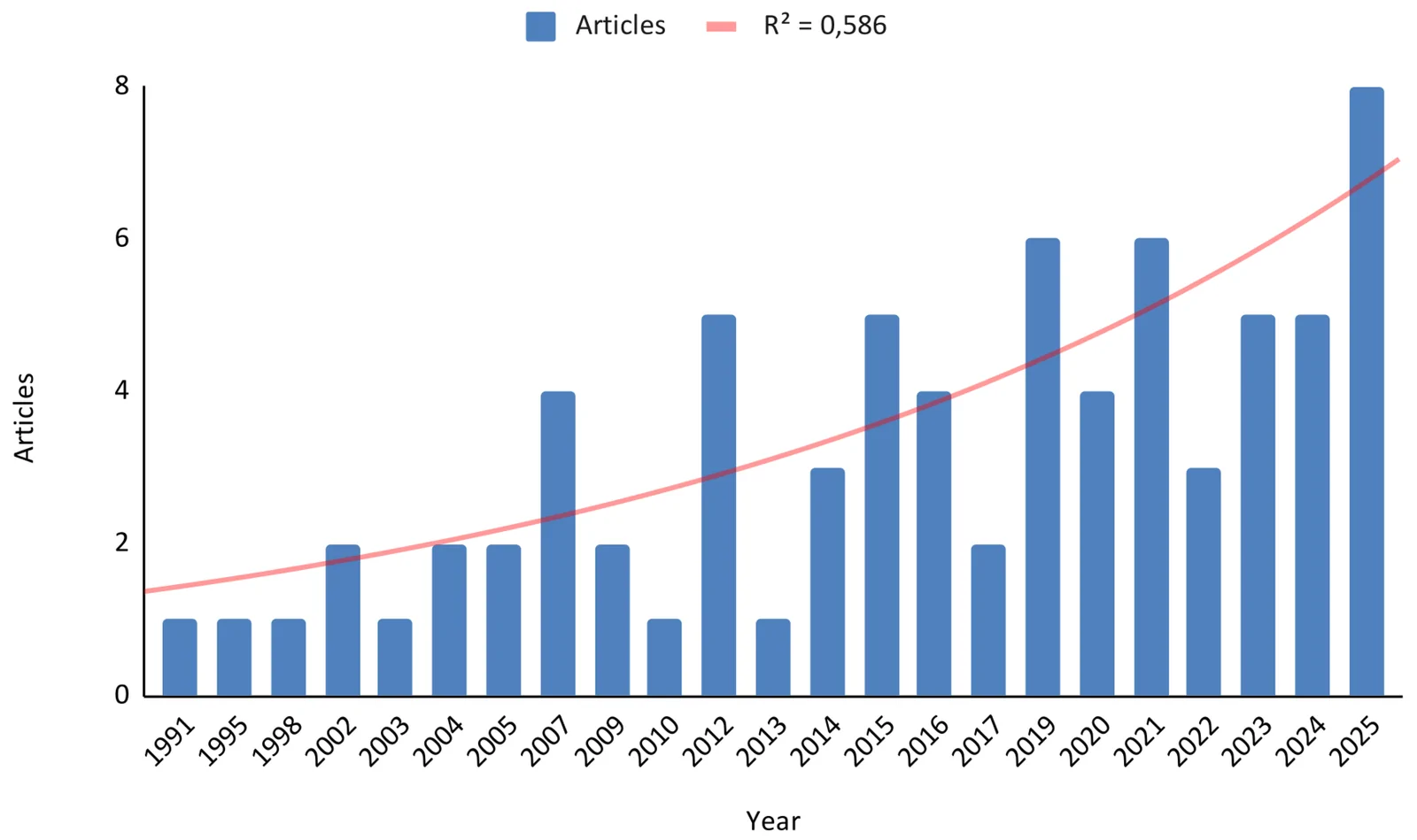
Publications and trends in the number of articles published over the period analyzed.

Publications on this topic have varied over the years, with an average (during the period analyzed) of three articles published per year (Figure 2). Starting in 2019, the volume of publications increased, with the average number of publications during that period approaching five articles per year. Despite the low cumulative number of publications—75 in total—studies on this topic have been published since 1991, confirming the interest in the use of biosensors for groundwater monitoring. The first study cited deals with the detection of the herbicide atrazine using a heterogeneous enzyme immunoassay. Between 1991 and 2000, 3 articles were published; from 2001 to 2010, there were 14; from 2011 to 2020, there were 38 in total; and in the last period (up to 2026), 28 were published.

**Figure 3:**
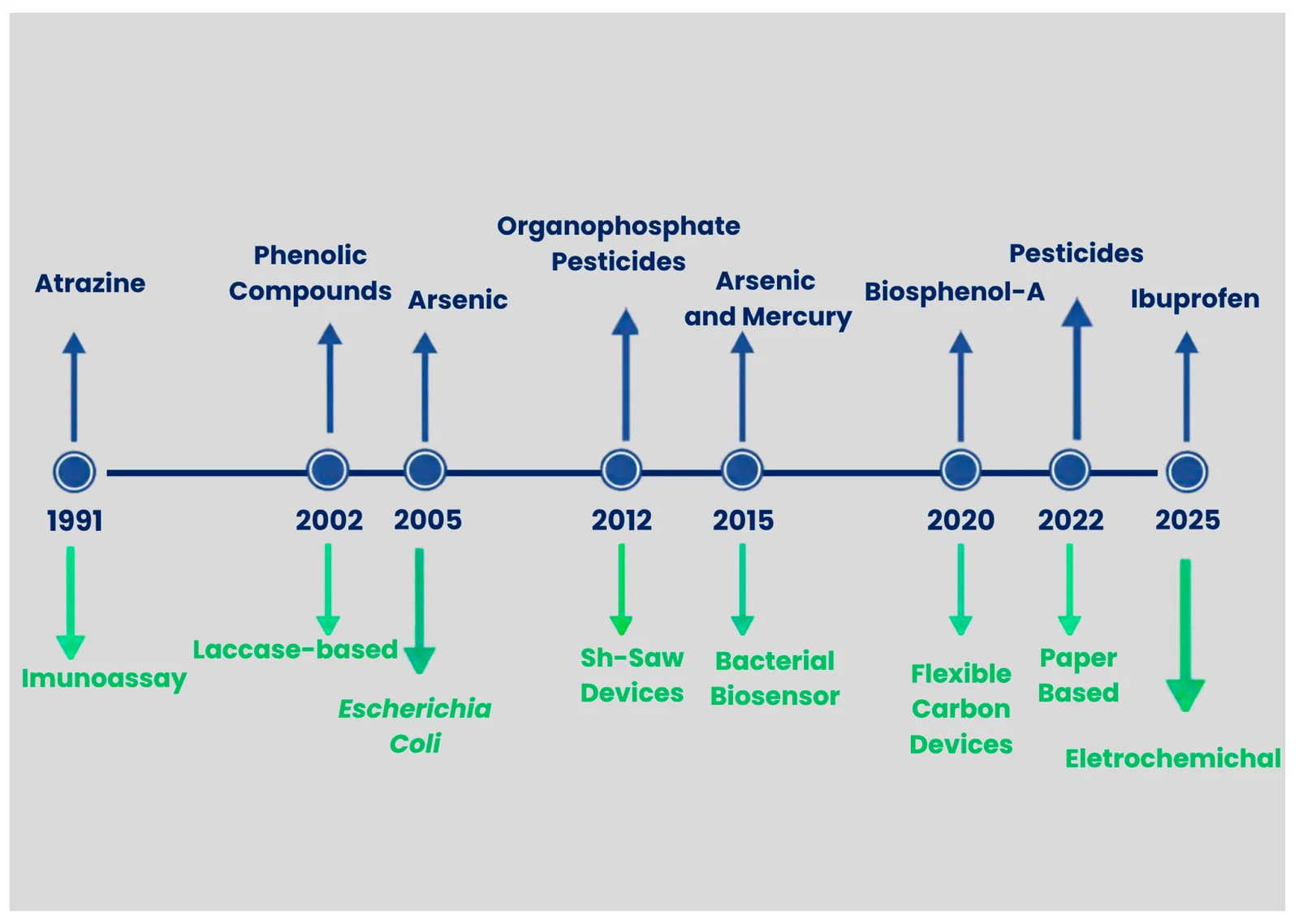
Timeline of some substances evaluated in the database articles and the technologies involved in their detection.

Figure 3 also presents a timeline (developed by the authors) of certain technologies used over the years, as well as the substances analyzed in groundwater. It highlights only selected years and substances of interest to demonstrate how the technologies employed have evolved over time, alongside the increasing diversification of the substances analyzed. This timeline was constructed based on an analysis of the articles, evaluating the publication date, the substance assessed, and the technology employed.

### 3.1. Most-Cited Articles

The ten most-cited articles are shown in Table 1. Publication dates vary, ranging from the beginning of the analysis (1991) to more recent periods (2022). However, it can be observed that certain topics receive more attention or are highlighted more prominently during different periods. Articles on arsenic were published between 2005 and 2007, while those dealing with antibiotics (tetracycline) are from more recent years (2014 and 2015). Both the year of publication and the interest in the topic—as represented by the number of citations—show that the pollutants of interest change over time.

The articles that received the most attention after 2020, with the highest number of citations, focus on the detection of substances found in pesticides (A paper-based electrochemical device for the detection of pesticides in aerosol phase inspired by nature: A flower-like origami biosensor for precision agriculture) or on substances widely used in the chemical industry (Flexible Carbon Electrodes for Electrochemical Detection of Bisphenol-A, Hydroquinone, and Catechol in Water Samples).

**Figure 4:**
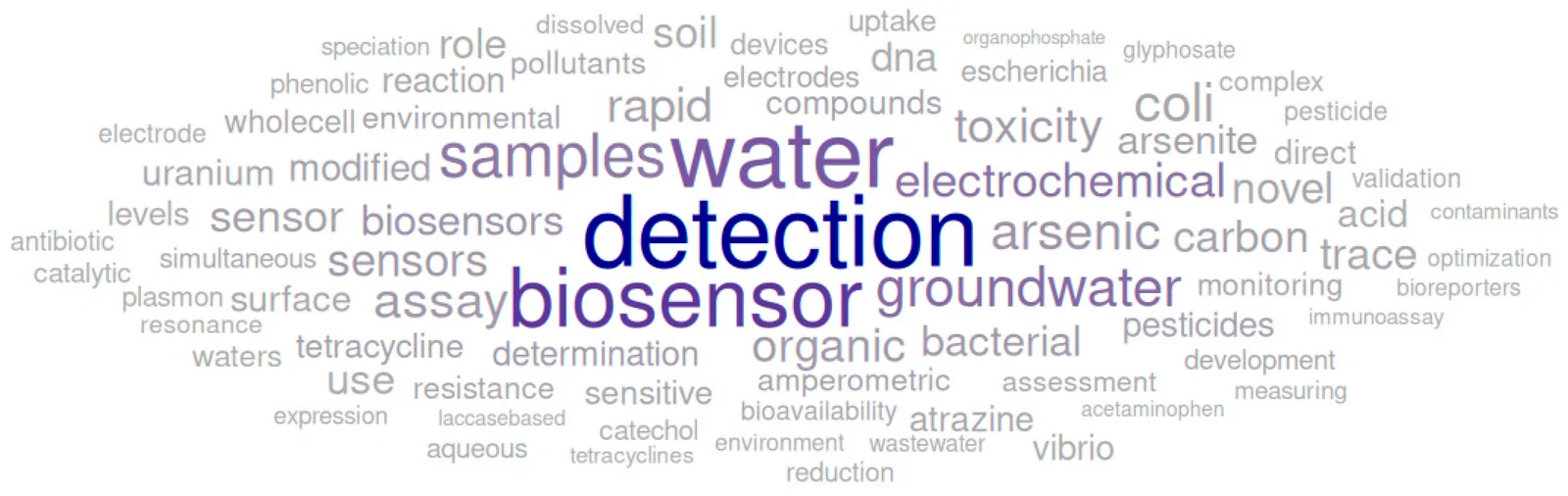
Word cloud of the titles of the analyzed articles.

When considering the most frequent words in article titles (Figure 4), the pollutants with the highest occurrence are: arsenic (6), arsenite (4), pesticides (4), tetracycline (4), and atrazine (3) (Table 2). Thus, in addition to being compounds that were the focus of the most-cited articles, they were also those with the highest number of publications, accounting for 28% of the articles.

Given that the title must be a precise synthesis of the text [36], and serves as the initial point of contact for editors, reviewers, and readers [37], it can be used for co-word analysis. Such networks can act as semantic proxies for conceptual relationships, helping to represent the cognitive content of scientific discourse [38]. Accordingly, the analysis described here utilized titles to identify the topics of interest among authors publishing on the subject. The analysis does not aim to compare time periods (due to variations in publication volume across periods), but rather to illustrate the themes under study.

In the analysis of keywords, the pollutants that appear most frequently are pesticides, cited five times in the singular and four times in the plural. An analysis of the article texts reveals that the substances most frequently analyzed are arsenic and agricultural chemicals.

Substances used in agricultural practices, such as herbicides and pesticides, appear in research from the beginning of the analysis (1991) through recent years (2026). The latter article deals with the identification of methyl parathion (MP), a highly toxic organophosphate insecticide widely used in agriculture.

Table 2 shows the frequency of appearance of the evaluated substances within the 80 most frequently occurring words found in the titles. The term “detection” is the most frequent, appearing 28 times (representing 4.075% of the total 80 words). Detection refers to the act or process of discovering, ascertaining, or identifying something that was previously hidden, subtle, or imperceptible. Rebollar-Pérez et al. [39] help clarify the usage of this term in the articles by stating: “In this work, a colorimetric assay based on decrease in catalytic activity of chloroperoxidase (CPO) enzyme has been developed for the detection and quantification of Organophosphorus Pesticides.”

In this context, the authors indicate that the developed sensor must initially signal the presence of the pesticides. Subsequently, it must also measure the amount of the pollutant present in the water (quantification). Therefore, this distinction must be taken into account when differentiating the objectives of each study analyzed. Studies indicating the detection of the substances in question primarily sought to determine the presence or absence of the substance, with quantification as a secondary objective. In contrast, studies that included the word “measurement” in their titles aimed to quantify the substances from the outset.

### 3.2. Biological Elements Used in Biosensors

The most commonly used biological elements are bacteria (*E. coli*, Caulobacter, etc.), fungi, nematodes, embryos, biofilms, or whole-cell systems (whole-cell/bioreporters). These elements appear in the titles of 19 articles (25%), with *Escherichia coli* being directly cited in three of the articles and used to analyze the presence of antibiotics in the samples of interest. Another bacterium mentioned is Caulobacter crescentus, in this case related to the detection of uranium. Both bacteria are widely used in microbiology; however, Caulobacter crescentus is naturally found in freshwater environments and those with low nutrient levels.

The bacteria *Pseudomonas putida* and *Vibrio fischeri* are mentioned in an article evaluating the toxicity of a pesticide. These two bacteria are used in environmental ecotoxicity tests; *P. putida* (a soil bacterium) is used to measure growth inhibition, while *V. fischeri* (a marine bacterium) is used to measure bioluminescence inhibition when exposed to toxic compounds such as pesticides and heavy metals.

The bacterium *Vibrio natriegens*, on the other hand, is cited in the analysis of petrochemical contamination. Because it is a marine bacterium, it adapts well to saline environments and grows rapidly. The widespread use of bacteria in the analyzed articles, as well as the variety of species, is explained by their ability to interact with target substances and produce specific, detectable signals in response to the presence or concentration level of the substance of interest [40].

### 3.3. Publications by Country

When analyzing the number of publications by country, there is no absolute concentration in any single country. Nevertheless, articles published by institutions in the United States of America (USA) account for 13.33% of the publications. However, China and India each account for 8% of the publications, Mexico for 5.3%, and Italy for 4%. These countries contributed 38.6% of the articles analyzed here.

Regarding the level of development of the countries, publications come from institutions in both developed and developing countries. Among the ten countries with the most publications, there are articles published in Brazil, India, Mexico, the Czech Republic, and Albania, as well as in China, the U.S., Italy, France and Denmark. However, although there is no clear concentration of publications in either developed or developing countries, there is a concentration of publications in European countries. If we analyze the 15 countries that published the most articles, seven of them are European countries.

When analyzing the articles’ keywords and the continent of the publishing institution, we note that among articles published in the Americas, uranium contamination receives the most attention (10 articles). Meanwhile, in Europe, arsenic is more prominent (8), and it is also the most-cited pollutant in articles published by Asian institutions (8). Although it is not an emerging pollutant, arsenic receives attention due to its co-occurrence with various emerging contaminants [41].

As for substances related to agricultural practices, they rank sixth in terms of the number of occurrences among the keywords in articles published in the Americas. Among Asian countries, substances used in agriculture do not stand out, with the term “pesticides” appearing in only two articles. In Europe, however, these substances rank as the second-most cited, with the term “pesticides” used in six articles.

### 3.4. Types of Biosensors Used

Electrochemical biosensors are the most widely used, being directly mentioned in the titles of 19 articles (25.33%). This type of biosensor was cited in a large number of articles related to the detection of herbicides and pesticides; of the total 19 articles (25.33%), eight were focused on the detection of contaminants resulting from agricultural practices, with the substances cited including, among others, simazine, methyl parathion, trichlorfon, and paraquat (Table 3). The term “pesticides” was mentioned three times in general, and the word “herbicide” appeared twice in the titles.

Electrochemical biosensors use biological receptors that respond selectively to specific targets; a typical configuration of this sensor involves the integration of these receptors with electrochemical transducers [9]. This type of biosensor does not require intermediate components for signal evaluation, as it directly converts a chemical reaction into an immediate digital electrical signal. Optical sensors are cited less frequently; for example, in groundwater analysis, samples may be turbid, contain high levels of silt, or other components that hinder sensor readings. In contrast, electrochemical sensors exhibit greater tolerance to complex matrices, as noted by Venegas et al. [42]. These authors observed a smaller signal attenuation in complex matrices with higher organic load and turbidity.

**Table 3:** Types of biosensors, limit of detection, selectivity, stability, shelf life, and recovery in real samples.

Types of Biosensors	Limit of Detection	Linear Range	Sensitivity	Substance	Reference
Electrochemical	0.00224 µg·L^−1^	0.01–10 µg·L^−1^	1.18 µg·L^−1^	Chlortoluron	[43]
Optical	0.2 μM	0.2–1 μM	0.82 μM	Terbufos	[39]
Photobioelectrochemical	0.002 μM	0.005–10.0 μM	0.7 μA cm^−2^ μM^−1^	Simazine	[44]
Optical	10 μg L^−1^	10–500 μg L^−1^	-	Arsenic	[45]
Electrochemical	1 ppb	1–500 ppb	1.43 ± 0.08 nA/ppb	Arsenic	[29]
Optical	10 μg L^−1^	5–100 μg L^−1^	7.5 μg L^−1^	Arsenic	[26]

Although electrochemical sensors offer some advantages, such as those mentioned above, ensuring reproducibility in complex matrices remains a significant challenge [42]. Regarding optical sensors, one of the advantages attributed to these biosensors is their low cost, for the arsenic biosensor your cost is ∼1 USD per sample compared with that of physicochemical methods (e.g., ∼50 USD per sample) [42]. Using this sensor, it was possible to categorize water wells as safe or unsafe based on the WHO (World Health Organization) guideline value for arsenic in drinking water (10 μg/L) [26].

Some analyses regarding the use of optical biosensors for water monitoring (in general) point to limitations such as the challenge of device miniaturization, ensuring the stability of the biological element, and achieving sensor reusability [46].

### 3.5. Groups of Articles

**Figure 5:**
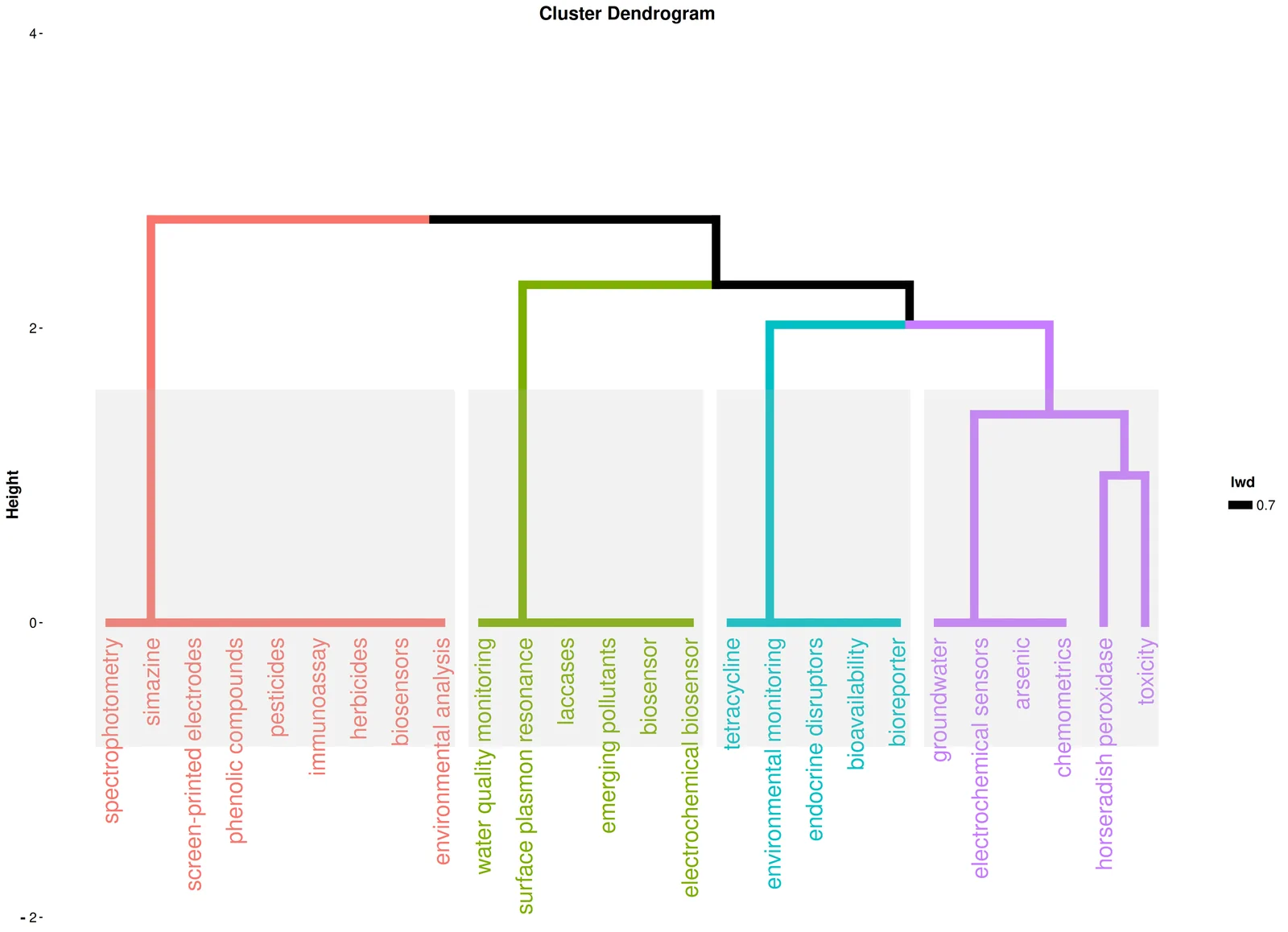
Cluster of related term groups found in the keywords.

By analyzing the themes of the articles using keyword co-occurrence, four major groups can be identified in the analyzed database (Figure 5). Keyword co-occurrence analyzes how these words are cited by authors, the frequency with which they appear in the analyzed articles, and with which other words they are cited; thus, it is possible to assess how different themes relate to one another in the articles and which theme has the strongest association with the cited word. Of the groups analyzed here, one relates to herbicide detection, another focuses more on electrochemical biosensors, a third focuses on tetracycline, and a fourth group focuses on arsenic detection.

In the first group, studies analyzing water contamination by herbicides and pesticides—such as simazine—are highlighted. This substance is a selective herbicide belonging to the triazine chemical group, used primarily in pre-emergence for weed control. This substance has received attention at different times, with articles published in 1991 and 2025, and in different countries (Germany and Brazil).

The group focused on electrochemical biosensors addressing emerging pollutants in general and water quality analysis. A key feature of this group is the use of laccases, a component that is immobilized on the sensor and oxidizes the target analyte. This oxidation generates an electric current, which is then converted into an output signal. Because it is an enzyme with high selectivity, simple operation (it does not require complex reagents for the reaction to occur), and because it produces only water as a byproduct of oxygen reduction, it is widely used in electrochemical sensors.

The third group focuses on the detection of tetracycline, a broad-spectrum antibiotic used to treat bacterial infections. Since the human and animal bodies do not fully metabolize this drug, its residues are excreted in urine and feces and are ultimately transported through sewage systems. This fact explains the presence of the term “endocrine disruptors” in relation to this drug in the evaluated group.

The last group, which relates to arsenic detection, includes terms such as electrochemical sensors, horseradish peroxidase, and, more closely related, the use of chemometric techniques. Chemometrics is the science that applies mathematical, statistical, and computational methods to extract as much useful information as possible from chemical data; in the database analyzed here, it was applied to the detection of arsenic in groundwater samples.

Horseradish peroxidase (HRP) is a metalloenzyme extracted from the roots of the wild horseradish plant (Armoracia rusticana). It is widely used in biochemical and biotechnological assays due to its high stability, high turnover rate, and ease of conjugation. In biosensors, it can be used both to detect pollutants that serve as its substrates and to monitor substances that inhibit its catalytic activity [47]. For pesticide detection, for example, the biosensor is exposed to a fixed amount of HRP and H_2_O_2_. When the contaminated environmental sample is added, the sensor’s response (electrical current or optical signal) decreases. The weaker the signal generated, the higher the concentration of pesticide in the sample.

### 3.6. Topics Discussed in the Articles over the Years

An analysis of the articles’ keywords reveals that certain topics have appeared more frequently in the studies analyzed. In 2025, the term “Nonsteroidal Anti-Inflammatory Drugs (NSAIDs)” appeared for the first time. NSAIDs are medications that relieve pain, reduce inflammation, and lower fever. They work by blocking enzymes that produce chemicals that trigger inflammation in the body; ibuprofen is an example of this type of medication. Ndreka et al. [48] note that from 2019 to 2025, there was an increase in researchers’ interest in the presence of these pharmaceutical products in water, which explains the emergence of this term in more recent years and the trend toward more frequent research on these substances in the coming years.

Some more recent studies also examine substances used for applications such as herbicides or pesticides but that were not mentioned in previous studies. One example is buprofezin, a growth-regulating insecticide and acaricide that acts as a chitin synthesis inhibitor. Although it has low toxicity to humans and other mammals, it is highly toxic to aquatic organisms and non-target insects. This insecticide was developed in the 1980s [49] and acts through direct contact with the insects’ bodies [50]. Its use, either in combination with other substances or on its own, is highly toxic to fish [51], which highlights the risks of contamination in aquatic environments. Furthermore, its presence in groundwater can lead to contamination of irrigation sources, resulting in the death of insects beneficial to agriculture and accumulation within the food chain.

The analysis of the themes over the years does not aim to compare the periods, but rather to show when the topics begin to emerge in the articles analyzed. Therefore, the publications are cited within each period rather than in comparison to the others.

When analyzing how the topics evolved over time, from 1991 to 2000, the three published articles had in common the use of immunoassays and immunochemical analysis. These analyses are laboratory tests that indicate the presence or quantity of a specific substance in a liquid sample (such as water) by utilizing the natural reaction between an antigen (the target) and an antibody.

Subsequently, between 2001 and 2010, toxicity analysis of certain substances came to the fore, with arsenic receiving the most citations (1.35% of the total keywords cited in the articles, being the first substance analyzed to appear in the ranking). Other relevant substances included atrazine; among the biological components of biosensors, bacteria were the most frequently cited during this period.

Between 2011 and 2020, arsenic continues to be the most-cited substance during this period (1.65%), with benzene also gaining prominence (0.96%). During this period, *E. coli* emerged as the most commonly used biological component (the first biological element to appear in the keyword ranking, with 1.03% of the total). The period from 2021 to 2026, despite being the shortest time span, accounts for 28 published articles. During this period, electrochemical sensors gained greater prominence (0.76% of the total keywords, making it the first type of biosensor to appear in the ranking), being cited more frequently than other types of sensors. No single substance stands out as the primary focus of detection; however, some pharmaceuticals begin to gain prominence, such as acetaminophen (paracetamol). Arsenite continues to be cited, and other types of substances—such as those resulting from agricultural activities—are cited in smaller numbers (one article each).

### 3.7. Types of Samples

An analysis of the types of samples used in the articles (Table 4) shows that laboratory-simulated samples account for the majority of the total (37, representing 49.33%). This type of sample consists of mixtures of deionized water and chemical reagents used to test the sensitivity of biosensors. Other substances that simulate groundwater are also used, as well as databases for simulations.

Samples collected directly from groundwater sources account for 24% of the total, while articles that use different water samples (not exclusively from groundwater) or use water from other sources to simulate groundwater conditions account for 26.66%. The prevalence of samples obtained in the laboratory highlights a limitation of the studies analyzed, which reinforces some of the discussions presented in the corresponding section regarding the lack of use and difficulties associated with the large-scale application of these sensors.

## 4. Discussion

Bacteria are the most commonly used biological component in biosensors. In particular, *Escherichia coli* has been applied in various pollutant detection systems. This bacterium is widely used in biosensors because it is easy to culture, which simplifies its use. Its growth is characterized by a shorter doubling time compared to other bacteria [52]. It is easy to culture and has lower nutritional requirements; furthermore, its genetics are well established, allowing it to be genetically engineered to produce mutants with desired characteristics [53].

Furthermore, parameters such as bioavailability, toxicity, and genotoxicity can only be tested using whole cells [54]. These authors emphasize that bioavailability and toxicity are important factors for detecting the presence of pollutants; consequently, the development of whole-cell biosensors has been directed, to a large extent, toward environmental applications. However, the use of these cells faces certain challenges, such as the stability of the cells employed, regulatory restrictions, and the need to optimize response times so that they can serve as a competitive detection tool in the market [55].

Enzymatic biosensors offer advantages such as scalability and sensitivity, enabling them to detect analytes even at low concentrations [56]. However, they have limitations, such as susceptibility to degradation, a factor that affects the performance and longevity of biosensors. In the case of immunosensors, this type of biosensor offers greater accuracy, specificity, and sensitivity than traditional methods [56].

It is not easy to determine which type of biosensor (or biological element) is most suitable for each type of contaminant. This is because, in addition to the small number of articles analyzed (75) for each type of pollutant, different detection methods are cited. For example, in a review on the detection of pesticides in aquatic environments, ref. [56] evaluated four types of biosensors (Aptasensor, enzymatic, whole-cell, and immunosensor) and found no concentration of just one type. In this analysis, the authors identified 27 different types of pesticides, which may explain the variety of sensors used. Each of these substances has different applications, uses, and modes of action, which also results in different behaviors of the biosensors in each case.

With regard to emerging pollutants, contamination caused by the presence of pharmaceuticals can be detected through the expression of genes in bacteria that are associated with the presence of these substances. Zhang et al. [34] observed an increase in the expression of the antibiotic resistance gene in *E. coli* when the concentration of tetracycline in the water was increased. The first member of the tetracycline family was discovered in 1945 by Benjamin Duggar; this group of antibiotics has a broad spectrum of activity, low toxicity, and low cost [57]. Because of these properties, they are widely used, which has led to the emergence of resistance in a wide variety of bacteria [58].

Jiang et al. [59] observed that a bioreporter using *E. coli* exhibited a dose-dependent response to mitomycin C, an antineoplastic antibiotic derived from the bacterium *Streptomyces caespitosus*. Used in the treatment of certain types of cancer [60], it is present in lower concentrations than other substances in water treatment systems; however, it has attracted attention due to its increasing use [61].

Traditional contaminants, such as arsenic, can also be detected using biosensors composed of bacteria. In this regard, the first biosensors to use whole cells to detect arsenic were developed by Corbisier et al. [62] and utilized genes from the *E. coli* Plasmid R773, which confers resistance to arsenic on the bacterium. Huang et al. [44] used the recombinant arsR-lacZ gene from a nonpathogenic strain of *Escherichia coli* DH5α and developed a color-based biosensor that could be observed with the naked eye or measured by a spectrometer.

Although arsenic is a naturally occurring element found in the environment, it is considered a human carcinogen and was ranked first on the list of hazardous substances by the Agency for Toxic Substances and Disease Registry (ATSDR) in 2013 [63]. Contamination by this substance, which affects aquifers, is related to geographic characteristics and mining activities. The most severe pollution is found in countries in South and Southeast Asia [64]. This fact corroborates the results presented here, as in Asian countries, arsenic was the most frequently cited contaminant in articles published on that continent. Arsenic is also found in conjunction with other contaminants [27]. Although they discuss the presence of arsenic alongside various emerging pollutants, the authors note that research on this type of co-occurrence has not yet yielded a significant number of published articles. They emphasize that greater attention is needed to analyze the co-occurrence of these contaminants, with a focus on those that possess endocrine-disrupting properties, such as pesticides, phthalates, etc.

The prevalence of publications in the U.S. stems from various reasons, one of which is the prevalence of scientific journals on biosensors published in that country [25]. Another point worth noting about the country is its funding network (public and private) and the role of U.S. universities in the development of technology in various fields [65].

The prevalence of publications on uranium contamination of groundwater in the Americas is due, among other factors, to the legislation governing this issue. Legislation such as the Clean Air Act (CAA) and the Safe Drinking Water Act (SDWA) regulates the ways in which uranium can be ingested by humans [66]. Amendments to these laws governing uranium were published in the 1980s and 1990s. Balaram et al. [67] report that high concentrations of uranium have been detected in water samples in countries such as the United States, Canada, South Korea, and China. These findings also help explain why interest in developing biosensors for detecting this substance is particularly strong in the United States.

On the other hand, countries in the Americas account for a large share of the use and approval of chemicals for agricultural purposes. About half of the pesticides approved for use in Latin America are not permitted in the European Union [68]. As discussed earlier, because it is one of the main sources of water for agricultural irrigation, groundwater used for this purpose is also more susceptible to contamination by pesticides and herbicides. Whether through rainwater runoff, soil infiltration, or direct contamination, countries in the Americas (particularly in Central and South America) are subject to higher rates of contamination, and thus the issue of detection using biosensors takes center stage.

In this thematic, most biosensors developed to detect presence of herbicides are based on measuring the inhibition of photosynthesis, utilizing biological elements such as chloroplasts [43], as in the study conducted by Dias et al. [45]. Despite this, these biosensors may exhibit low specificity, detecting different types of herbicides [45]. Immunosensors, which combine the affinity of monoclonal antibodies with the sensitivity of optical or electrochemical detection, may serve as alternatives for this purpose [43,69].

The detection of residues from human activities in groundwater is primarily carried out using electrochemical biosensors. This type of biosensor was developed because it is efficient and fast, in addition to being environmentally friendly [70]. In this type of biosensor, the electrode serves as the element used to perform biotransduction [71]. Electrochemical biosensors utilize a biological recognition component in conjunction with an electrochemical transduction element to obtain quantitative or semiquantitative analytical measurements, according to Bindu et al. (1995), as cited in [72].

In the studies analyzed here, biosensors of this type were used to detect: bisphenol-A, hydroquinone, and catechol [35]; methyl parathion [73]; trichlorfon [74]; paraquat [75]; pesticides [32]; herbicides [43]; and other substances. This widespread use is due, among other factors, to the sensitivity and selectivity of this type of biosensor in the presence of the substance of interest, as well as its low detection limits [76]. Initially, the demand for these sensors stemmed from their speed of analysis, low cost, and selectivity; currently, there is a demand for the development of systems capable of handling complex samples, suitable for field deployment, and capable of detecting substances in the presence of others [77]. The potential for interference due to the presence of other substances in water samples, durability, production costs, and the need for constant calibration are limitations that should be addressed in future research [76].

In addition to the various agricultural activities that can contribute to groundwater contamination, these areas are also subject to the disposal of agro-industrial waste [78]. Managed Aquifer Recharge (MAR) is an essential practice for groundwater conservation and sustainability, as well as for increasing drought resilience, particularly in arid and semi-arid regions [79]. In areas where agricultural practices are carried out, beyond contamination from waste generated by these activities, the combined effects of heavy metals, nitrates, pH variations, and pharmaceutical pollution must be considered [79]. The presence of these various contaminants interferes with the results obtained by biosensors when tested with real groundwater samples, representing a limitation in some of the studies presented in this review.

Although mentioned as an alternative for sample collection, the use of smartphones is not discussed extensively in the articles analyzed here. Only two articles (2.66%) mention the use of this device. The two uses cited are as a potentiostat, for data visualization, and for capturing fluorescent images. In the field of health, Beduk et al. [80] found more studies addressing the combined use of biosensors and smartphones. They identified studies with a variety of applications, ranging from measuring glucose levels to detecting viruses. Since the term “smartphone” was not included in the search, the small number of articles on its use may be due to this fact. Savas and Gharibzahedi [81] point out that the use of smartphones in conjunction with biosensors, leveraging the increased processing power of these devices, can facilitate the analysis of larger volumes of data, thereby enabling the analysis of more complex samples.

Indeed, a significant gap to be addressed in research on this topic is the development of biosensors capable of detecting more than one substance, thereby enabling the assessment of simultaneous occurrence and cross-contamination by different pollutants. In the case of emerging pollutants—which occur at lower concentrations than some traditional contaminants—such detection is essential to prevent the presence of these substances in groundwater from being masked or underestimated.

## 5. Limitations

The limitations of this study are linked to the limitations of the databases used to retrieve the articles. Every database has limitations regarding the number of indexed journals, a figure that also fluctuates over time. Furthermore, using English as the standard for article selection may exclude works published during periods when English was not the standard language of publication, or works from countries where research (or at least the abstract) is not published in that language.

Adopting a bibliometric approach also imposes certain limitations on specific assessments, such as the analysis of word co-occurrence in titles, keywords, and abstracts. Although the analysis (following an initial review of these fields) was based on reading the articles (particularly the methodology sections), there remains a risk of author bias, as specific aspects of the articles may have been emphasized at the expense of others.

Conducting future research using a meta-analysis of the articles, hereby enabling the calculation of an overall effect or general pattern of evidence, could yield further insights into the efficiency of different sensors in analyzing specific substances. However, this would require a larger dataset than the one evaluated here.

## 6. Research Advances and Innovations

Despite the limitations of the research and the methodology adopted, the study succeeded in advancing the systematization of knowledge on the subject. This distinguishes it from other existing reviews, which tend to aim for a general description rather than a systematization. Furthermore, the use of clustering makes it possible to identify groups of interest within the field, thereby helping to map existing knowledge and areas of future trends. The study presented here also helps identify research gaps—specifically, the need to evaluate the co-occurrence of different pollutants and its impact on the performance of the tested biosensors. This may well be the article’s major contribution to discussions in the field and to the development of future research.

## 7. Conclusions

In conclusion, based on the questions that guided this review, the biological elements most frequently cited in the monitoring of emerging pollutants in groundwater were bacteria, with particular emphasis on the use of *E. coli* in various applications. A recent application of these organisms involves assessing the presence of antibiotics in groundwater samples, a type of pollution that has received increased attention since 2019. Despite this, bacteria or whole cells are also used to monitor traditional contaminants, such as hydrocarbons or arsenic.

Arsenic was mentioned in various studies and was one of the pollutants that received the most attention in the evaluated research. In the context of groundwater, this is because arsenic is frequently found in the analyzed samples, with such contamination occurring through natural processes or mining activities. However, there are still significant gaps to be filled in the analysis of this contaminant, particularly regarding its interaction with other substances.

As for the types of biosensors, electrochemical biosensors are the most frequently cited, with applications for detecting various contaminants. Despite their low cost, ease of use, and ease of interpretation, the limitations noted regarding these biosensors relate to the difficulty of analyzing results from samples containing more than one substance, the need for large-scale production, differences in results between laboratory and field tests, and the need to develop systems with greater sensitivity for lower concentrations.

## Figures and Tables

**Table 1:** The analyzed database.

Paper	Total Citations Through 2026	Total Citations per Year	Normalized Global Citations (NGCS) ^1^	Reference
		(Year of Article Publication Through 2026)		
Trang P, 2005	139	6.318	1.972	[26]
Chen Z, 2015	103	8.583	2.960	[27]
Zhuang J, 2013	95	6.786	1.000	[28]
Male K, 2007	92	4.600	2.258	[29]
Song Y, 2009	82	4.556	1.333	[30]
Smirnova I, 2004	80	3.478	1.600	[31]
Caratelli V, 2022	70	14.000	2.442	[32]
Krämer P, 1991	64	1.778	1.000	[33]
Zhang Y, 2014	63	4.846	1.331	[34]
Sá A, 2020	56	8.000	1.445	[35]

^1^ NGCS: Total citations for the article divided by the average number of citations for all articles published in the same year.

**Table 2:** Frequency of the analyzed substances that appear most often in article titles.

Word	Number of Occurrences	Frequency
arsenic	6	0.873
arsenite	4	0.582
pesticides	4	0.582
tetracycline	4	0.582
atrazine	3	0.436

**Table 4:** Types of samples used in the articles analyzed.

Types of Samples	Total of Papers
Simulated laboratory sample, simulated data or deionized water.	37
Groundwater samples	18
General water samples (tap water, river water, etc.)	20

## Data Availability

The data from this article can be accessed at https://doi.org/10.17605/OSF.IO/95QBS.
